# Traumatism as a Factor in Ovarian Pathology

**Published:** 1894-03

**Authors:** James Kennedy

**Affiliations:** Professor of Pharmacy, Etc., Texas Medical College, Galveston


For Texas Medical Journal.

### TRAUMATISM AS A FACTOR IN OVARIAN PATHOLOGY.

BY JAMES KENNEDY, M. D., PH. G.,
Professor of Pharmacy, etc., Texas Medical College, Galveston.

THE subject of traumatism as a factor in the production of pelvic lesions was forcibly brought to my notice some eighteen months ago, when I had occasion to operate upon a young lady for the removal of both ovaries.

The patient had been thrown from a car with considerable violence and disease of both ovaries supervened.

The case was reported in DANIEL'S TEXAS MEDICAL JOURNAL, upon the patient's recovery, but the legal features of the case have never been given to the profession. They are of such an interesting nature that I feel warranted in reporting them in this connection.

I will briefly summarize the facts by stating that two separate suits were instituted against the railroad company, one to recover expenses and the other to obtain damages for injuries received.

The corporation, against whom the suit was brought, tried to prove that the girl sustained no serious injuries, and any permanent injury or disability from which she suffered, was due to the treatment or operation; the latter being entirely unnecessary.

In producing their testimony the company placed upon the stand a distinguished physician, who testified, in the capacity of expert, that the operation for the removal of the ovaries in the case of this girl was not only wholly unnecessary but unjustifiable. This distinguished operator stated under oath that it was not possible for an accident of this character to produce the lesion diagnosticated by the operator and confirmed by the operation. He further stated, that inasmuch as the ovaries were the best protected organs in the body, they were entirely secure from the dangers of any traumatism which did not occasion a solution of continuity of the external structures. This opinion was shared by several other physicians of good attainments.

A statement so remarkable as the one quoted, and emanating from a man of reputation, merits the most serious consideration.

In the progress of the trial or suit it was proven that the health of the patient had always been good, and that no disturbance of the menstrual function was experienced until after the receipt of the injury. (For a detailed account of the case the reader is referred to the September number of the journal above quoted.) It was further shown that the constitution of the patient had always been of the most robust type. The girl's testimony and that of her physician showed that immediately after the receipt of the injury menstruation became irregular, and that the symptoms which are so characteristic of ovarian disease, supervened. Several consultants, including the authority referred to, were called, and all admitted the presence of a pathological process in the left ovary, but there were several who questioned the probability of a diseased process in the right ovary. Operation was decided upon by her physician and stoutly opposed by my distinguished consultant. Recovery resulted. The ovaries, when removed, showed evidence of degeneration, of the cystic variety. One ovary, the left, was found to be about four times the size of normal, whilst the right was increased in size, though not so large as the left.

As the joint suits involved thirty thousand dollars, the case was very vigorously contested, and, as may be imagined, the result was looked forward to with no little anxiety by the writer. Judgment was obtained in both cases, and while the amount was not that sued for, especially in the case of the damage suit, it decided the question of the justification of the operation and was a great satisfaction to the operator.

In this day of bacteriological enlightenment and research the causative factor in all manner of disease is said to be of microbic origin, and every other factor is made subservient to theories which make the germ all-powerful in the domain of pathology, without according to them their share of importance, or acknowledging their influence in the production of visceral lesions. It is not my design to lessen the importance of germ-influence, but to impress the importance of mechanical violence in the production of diseased processes in the ovaries.

I am well aware of the fact that there are many physicians and surgeons who are of the opinion that an inflammation of any viscus cannot exist without the presence of pus, and necessary pus-producing germs. I do not deem it expedient to discuss, in this connection, the essential feature of a true inflammation, but will here state that it is my opinion, which is based upon observed cases, that there are many lesions of the pelvic organs which are induced by traumatism and which are quite independent of pus-formation, For instance, in the case of the patient just referred to, structural changes of the parenchyma of the organs were produced by the violence, and suppuration, which is considered one of the essential features of inflammation, was entirely absent.

In the case of a married lady who had never borne children, and upon whom I had occasion to operate for the removal of the uterine appendages, some months since, I found the organs diseased in an exactly similar manner as the specimens already referred to. The history of her case showed that she had sustained an injury to the pelvic organs as the result of a fall received some years before. The adhesions were very firm and dense, showing that the inflammation process had existed for some time. The symptoms of disturbed nutrition supervened upon the receipt of the injury and increased in severity. On examination the ovaries were found somewhat enlarged in size and adherent by strong bands which bound them firmly in their unnatural position.

On their removal they were found to have suffered pronounced structural changes. I have had occasion to examine and treat a very considerable number of cases which presented similar histories, and in which examination showed that the organs had assumed a pathological condition, with no previous or concurrent condition of purulent inflammation. That is, in none of the cases to which I refer was there any leucorrhœa, or evidence of pus in the organs removed. It is my opinion that traumatism is capable of disturbing the nutrition of the pelvic organs in a manner precisely identical to that which is so manifest in other organs more superficially located, and that while in many cases germs find access to the structure whose vitality has been lowered by violence, their presence is not at all necessary to produce degenerative changes. I cannot agree with those who hold that *if it were possible for an ovary to suffer violence*, in the absence of germs, the nutrition of the organ would be restored spontaneously.

Cases which have come under my observation have convinced me that the following changes are produced in the healthy ovary as the result of violence: Hyperæmia, from the irritation, followed by congestion, transudation of serum, and, if the malposition favors, the formation of adhesions through the organization of the exudate. If conditions do not favor resolution, the serum, which is poured out into the Graaffian follicles, distends them to the extent of obliterating the parenchyma of the organ. There is to my mind no valid reason why the same changes which occur in the inflammation of the peritoneum in other parts of the body as the result of traumatism, should fail to obtain in the pelvic peritoneum. It is a patent fact that the ovaries and uterus may suffer displacement as the result of violence, that an ovary may be prolapsed or a ligament torn, and in such an event a pronounced disturbance of nutrition may result, producing the conditions to which I have just referred.

